# New York Letter

**Published:** 1881-02

**Authors:** Geo. B. Pratt

**Affiliations:** New York


### Article VIII.

New York, Jan. 3, 1881.

Editors Journal and Examiner:

Having said farewell for a few weeks to my pleasant Indiana home, and come East for the purpose of visiting the medical schools and hospitals of New York, Philadelphia, and Boston, I thought a letter of gossip from this city (and perhaps at another time from the other two as well), might not be without interest to your readers, many of whom can, no doubt, recall from the long ago pleasant memories of the winter months passed in one of these places, at the commencement of their medical career.

The three colleges here seem to be in an equally prosperous condition, each of them having over five hundred matriculants. At the College of Physicians and Surgeons, instead of a spring session, and a succeeding winter session of five months in length, which hitherto have made up the collegiate year, this now consists of a single session of somewhat over seven months in length. Bellevue has adopted the three years course, with final examinations in elementary branches at the end of the first and of the second session. The collegiate year at the University is divided into three sessions, the spring, preliminary winter, and regular winter session, the whole embracing a period of eight and a half months attendance at the regular winter session being obligatory.

Father Time has dealt kindly with most of the older members of the different faculties. Alonzo Clark (now president), as much a favorite with the students as ever, finds it less tiresome to deliver his lectures, seated, although his voice shows no signs of weakening. Alfred Post still conducts the clinic at the University, and operates with as steady a hand as of yore; while James R. Wood, having lost none of his daring and dexterity, fills the large amphitheater at Bellevue Hospital with students from all the colleges, who come together Saturday afternoons to attend his surgical clinics; and Lewis A. Sayre, whom many think gruff and unsympathetic, yet whose friends know the warm heart beating beneath the rough exterior, has for his audience, many practitioners from this and neighboring cities. At one of his clinics, having shown a case of hip-joint disease, he said: "Such cases as these are often attributed to scrofula, and I am tired of hearing everything laid at its door. Let me cite a case bearing on this point. I was called to see a lady, recently confined, who was suffering from some trouble with both hip-joints. It had been necessary to use the forceps in delivering the child, and the assistants holding the leg on either side, had caused such extreme abduction as to produce the affection of the hip-joints for which I was called in consultation. While explaining the modus operandi to the physician, the nurse, who was dressing the child, having become interested in my remarks, had taken the child by the legs and was making extreme abduction. I caught the little one's legs together, and said to the doctor, 'if this child shows no bad effects from that proceeding, I shall be surprised.' Not long afterwards, the child was brought to me with morbus coxarius—of scrofulous origin, of course. The mother had it, and now the child has it."

The elder Flint, though looking older than when I saw him last, is evidently in the best of health. H. B. Sands, now occupies the chair of Practical Surgery at the college of Physicians and Surgeons, and ranks among the most skillful surgeons in the city.

I have had the pleasure of witnessing several ovariotomies done by T. Gaillard Thomas, at the Woman's Hospital. In one of these the time occupied from the first incision, to the twisting of the last suture, was exactly fifteen minutes. The tumor was a large multilocular cyst, with a long pedicle, and no strong adhesions. The incision in the abdominal walls was made low down in the median line, and was not over three inches in length. Dr. Thomas said, "Every inch that the incision is extended toward the diaphragm, adds immensely to the dangers of the operation." The operation was done antiseptically, Dr. Thomas being a true disciple of Lister. James L. Little, formerly connected with the college of Physicians and Surgeons, is now Clinical Professor of Surgery at the University. At his last clinic he cut for stone, in a little boy about four years old, doing the median operation. Dr. Little stated that he had performed lithotomy thirty-six times, on patients old and young, and had removed some very large stones, but of these thirty-six cases only two had died, and their death was not directly attributable to the operation. He used the median operation, and thinks his results were as good, if not better, than would have been obtained from any other method.

At St. Luke's Hospital I saw a very interesting operation by Thomas T. Sabine, a detailed report of which will probably appear in the *American Journal of Medical Sciences.* A man, falling from a scaffold thirty feet high, alighted on his feet, and fractured the astragalus in the right tarsus. The fracture was not discovered at the time, but the man was treated for simple sprain of the ankle-joint. When he entered the hospital some months later, the foot was in a state of talipes varus, but the motions of the joint were quite good. After consulting with Drs. Sands, Mason, Wier, Post, Stimson, and others, Dr. Sabine decided to divide the tibia and fibula subcutaneously, at the junction of the lower and middle thirds, which he did very dexterously, losing but little blood. He then abducted the lower fragment to a sufficient angle, to bring the sole of the foot on a line at right angle with the axis of the leg, where he secured it by means of the plaster-of-Paris splint, strengthened by a strip of iron on either side. The interval between the ends of the tibia, at the point of division, was about three-fourths of an inch. Dr. Bull did a similar operation at the same hospital, a few weeks since, and the result was all that could be hoped for.

I was at the Woman's Hospital on one of Dr. Emmett's operating days, and, among other operations, saw him close a vesico-vaginal fistula. Completing the operation, he said: "Gentlemen, the reason why so many of these comparatively simple operations fail, is on account of the wires being twisted too tightly, and so quickly cutting their way out. Be careful, before commencing to twist, to shoulder or bend each wire at a point directly over the line of apposition; then, by bending both wires together, at nearly a right angle to this line, you can twist without danger of constricting the tender tissues." Dr. Edmunds also operated on a girl fourteen years old, for the formation of an urethra. Some surgeon, unknown to fame, in trying to remove a large calculus from this girl's bladder by dilating the urethra, had torn a rent from a point in the base of the bladder to the external meatus. So much time had elapsed, that not a sign of the position occupied by the urethra remained. The doctor said he would not attempt to close the whole rent at one sitting, but would first make the urethra, and later, close the opening in the base of the bladder. Dr. Emmett has had good results in this class of cases. He makes up for the lack of sphincter muscles, by extending the new urethra up in front of the symphysis, bringing the external meatus fully an inch farther forward than normal. This makes a urethra having a shape similar to the spout of a tea-pot.

Lister's method is the fashion here, but there is one professor who does not hesitate to express his views on the subject, notwithstanding they are not in accord with those of the majority of his colleagues. Alexander B. Mott, said to his class at Bellevue recently, "For twenty years I have been holding surgical clinics in this college, and feel confident that the results attending my operations, will compare favorably with those obtained by what is termed 'antiseptic surgery.' The great majority of you will settle in small places, where you would not find the necessary paraphernalia, and could not afford it if you did; and you will find by doing your work neatly, and using plenty of clean water, that ninety-nine out of every hundred of your surgical cases will do as well as you could expect from any treatment."

In closing, let me say a word or two regarding a lecture I attended a few evenings since. Mr. Henry Bergh addressed the citizens of this city on the subject of "Vivisection." That Mr. Bergh has done a good work in the prevention of cruelty to animals, no one will gainsay, but when he comes before an enlightened audience with such twaddle as he presented on this occasion, one not only perceives the gross ignorance of the man, but is led to doubt his soundness of mind. Hear him: "Of what use is vivisection? A friend of mine had a beautiful little daughter lying dangerously sick with diphtheria. The attending physician called in consultation a well-known physiologist of this city, who advised making an opening into the windpipe. The dear little girl screamed aloud at the horrible suggestion, and said: 'Father, they will kill me!' The operation was done; the child died, and her infantile prophecy was fulfilled. Had vivisection taught this man anything?" The argument is unanswerable. Comment is useless.

Recall to mind those eminent physiologists, whose arduous labors and patient researches have added so much to the sum of earthly happiness; whose whole lives were spent in the interest of their fellow men, and who went to their rest at last, recompensed by the thought of the good they had accomplished. And now listen to the tribute of respect paid them and their followers, by this champion of the brute creation. "If there is a hell, and hell-fires are burning, I am sure these men will feel their torments to the utmost extent." The long continued hissing that greeted this expression, must have startled somewhat this noble-hearted man, though we would not expect one capable of conceiving such a fiendish thought to be much disturbed. These extracts will suffice to illustrate the method by which Mr. Bergh hopes to convince the legislature of this State of the uselessness and wickedness of vivisection.

GEO. B. PRATT, M.D.

